# Comparison of Retinal Microvascular Vascular Density Between Adolescents With and Without Simple Myopia Using Optical Coherence Tomography Angiography

**DOI:** 10.5152/eurasianjmed.2023.22278

**Published:** 2023-02-01

**Authors:** Kemal Bayrakçeken

**Affiliations:** 1 Department of Ophthalmology, Erzincan Binali Yıldırım University Faculty of Medicine, Erzincan, Turkey

**Keywords:** Myopia, optical coherence tomography angiography, vessel density.

## Abstract

**Objective:**

The aim of this study is to investigate whether there is a difference in retinal microvascularization between adolescents with and without simple myopia using optical coherence tomography angiography.

**Materials and Methods:**

Thirty-four eyes of 34 patients aged 12-18 years diagnosed with school-age simple myopia (0-6 diopters), and 34 eyes of 34 healthy controls of similar ages were included in this retrospective study. The ocular, optical coherence tomography, and optical coherence tomography angiography findings of the participants were recorded.

**Results:**

The simple myopia group had statistically thicker inferior ganglion cell complex thicknesses compared to the controls (*P* =.038). The macular map values did not statistically significantly differ between the 2 groups. The foveal avascular zone area (*P* =.038) and circularity index (*P* =.022) values were statistically lower in the simple myopia group compared to the control group. The superficial capillary plexus outer and inner ring vessel density (%) (superior and nasal) showed statistically significant differences (outer ring superior/nasal *P* = .004/*P* =  .037; inner ring superior/nasal *P* = .014/*P* =  .046).

**Conclusion:**

Similar to high myopia, vascular density in the macula decreases as the axial length and spherical equivalent increase in simple myopia.

**Main Points**

- Optical coherence tomography (OCTA) has been used as a noninvasive technology that allows for the assessment of retinal microvascularity.
- In the literature, many studies have shown that high myopia causes vascular changes (especially in vessel density and foveal avascular zone parameters) in the retina. In this study, unlike the literature, vascular changes in the macular region of simple myopia were examined and compared with those of the healthy age-matched volunteers.
- Our results showed that there might be changes in the macula in simple myopia.

## Introduction

Myopia occurs as a result of several factors. There is substantial evidence of a link between myopia and prolonged close visual activity, particularly reading and personal computer use. The incidence of myopia is increasing in children and adolescents.^1,2^ A refractive error of less than −6 diopters is defined as simple myopia (school myopia), and a greater refractive error is defined as high myopia (pathological myopia). High myopia is characterized by progressive anteroposterior elongation associated with a series of secondary ocular changes.^2^ Many studies have demonstrated retinal vascular changes in high myopia. Using optical coherence tomography (OCT), it is now possible to examine the layers of the retina in detail and make quantitative evaluations. However, OCT does not provide information on the microvascular structure of the retina and choroid. Optical coherence tomography angiography (OCTA) devices, which have recently started to be used in daily practice, are very helpful in the detection and treatment of retinal and macular pathologies.^3^ Optical coherence tomography angiography is a very reliable and quick procedure that can be performed to measure retinal vessel density and foveal avascular zone (FAZ).^4^ It can be used when there is a need to obtain a detailed image of retinal vascularization. Previous studies have shown changes in retinal vessel densities in high myopia using OCTA compared to the general population, but to our knowledge, no similar study has been undertaken with school-age simple myopia.

The aim of this study was to investigate whether simple myopia (refractive error ˂ 6 diopters) differs from the general population by detecting changes in macular thickness and retinal vascular density using OCT (Nidek Co. Ltd., Aichi, Japan) and OCTA Nidek's RS300 advance (Nidek Co. Ltd., Gamagori, Japan) devices. We also aimed to determine whether retinal microvascular changes were directly related to the axial length (AL) and spherical equivalent (SE) in simple myopia.

## Materials and Methods

This retrospective research included 34 eyes of 34 patients aged 12-18 years with school-age simple myopia (0-6 diopters) and 34 eyes of 34 controls of similar ages with no known systemic or eye disease. Each patient underwent an ocular examination, visual acuity (LogMAR converted) measurement, and anterior segment and fundus examinations, including detailed refraction measurements. Cycloplegic refraction was performed and the measured myopia values (diopters) were recorded as SE. Axial length measurements were performed using the Nidek AL-Scan instrument (Nidek CO., Gamagori, Japan). Patients with any ocular disease other than myopia and those with a history of ocular surgery were excluded from the study. Patients with myopia with a refractive error higher than −6 diopters and hyperopic eyes more than 1 diopter were also excluded from the study. Ethical approval was obtained from the Clinical Research Ethics Committee of Erzincan Binali Yıldırım University (date: October 10, 2022, decision no: 03/10). Written informed consent was obtained from all participants who participated in this study.

**Figure 1.**
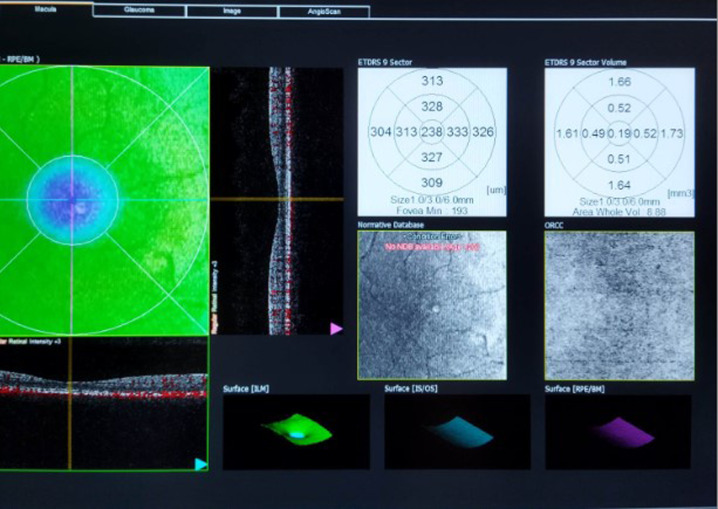
Measurement of the macular thickness according to the ETDRS chart using Nidek’s RS-3000 Advance and Navis Ex. Ver. 1.1.5 software. ETRDS, Early Treatment Diabetic Retinopathy Study.

**Figure 2.**
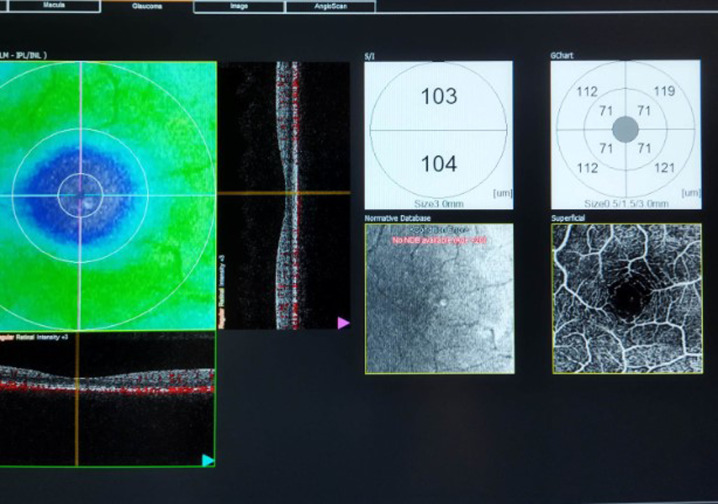
Superior and inferior ganglion cell complex thicknesses.

**Figure 3.**
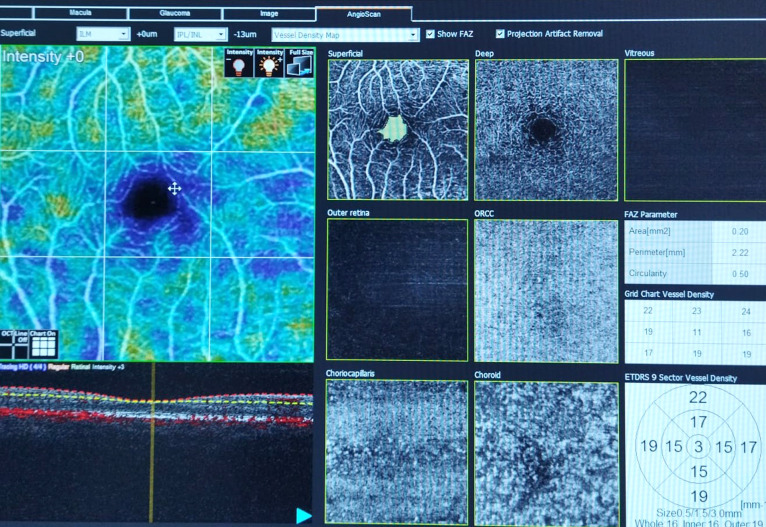
Foveal avascular zone metrics and vessel density values measured automatically by AngioScan software. The software only calculates the VD metrics at the level of the superficial capillary plexus. VD, vessel density.

The macular thickness [9 sectors defined by the Early Treatment Diabetic Retinopathy Study (ETDRS)] and ganglion cell complex (GCC) thickness were measured using OCT. In addition, FAZ parameters [area, perimeter, and circularity index (CI)] in the macula region and the vessel density (VD) values of the outer and inner part of the superficial capillary plexus (SCP) were measured using OCTA. The OCT and OCTA images of the patient and control groups were analyzed and recorded (Figures 1-3).

### Statistical Analyses

Data analyses were performed using Statistical Package for Social Sciences v. 23.0 software (IBM SPSS Corp.; Armonk, NY, USA). The descriptive statistics of the analysis results were given as numbers and percentages for categorical variables and mean standard deviation, minimum, and maximum values for numerical variables. The Shapiro–Wilk test was used to assess the compliance of the data with the normal distribution criteria. The comparison of numeric variables between 2 independent groups was undertaken using the Student’s *t*-test when the normal distribution condition was met, and the Mann–Whitney *U* test otherwise. The Spearman and Pearson correlation tests were used to analyze the relationship between numerical variables. The statistical significance level was accepted as *P* < .05.

## Results

There were 34 eyes of 34 patients in the simple myopia group and 34 eyes of 34 healthy volunteers in the control group. The overall mean age of the participants was 15.36 ± 1.77 years (range 12-18 years). Twenty (58.82%) participants were male, and 14 (41.18%) were female. There was no statistical difference between the 2 groups concerning the distribution of male and female participants and age (age/gender, *P* =.55/*P* =.22). Table 1 presents the participants' demographic data.

The ocular and OCT findings of both groups are shown in Table 2. There was a significant difference between the 2 groups with respect to the SE (*P* ˂ .001) and AL (*P* = .001) values. The mean superior GCC thickness was similar in both groups. However, the inferior GCC thickness of the simple myopia group was statistically significantly higher than that of the control group (*P* = .038).

There was no statistically significant difference between the 2 groups regarding the thickness (9 sectors) of the macular region according to the ETDRS chart (Table 3).^5^

Table 4 shows the OCTA findings of both groups. No statistically significant difference was observed between the 2 groups in terms of the FAZ perimeter, but the FAZ area (*P* = .038) and CI (*P* = .022) values were statistically significantly lower in the simple myopia group compared to the control group. A statistically significant difference was also observed found between the 2 groups in relation to the VD (%) values of the SCP outer ring (superior and nasal quadrants, *P* =.004 and *P* =.037, respectively) and SCP inner ring (*P* =.014 and *P* =.046, respectively).

The correlations of the OCTA findings with the SE and AL parameters are shown in Table 5. According to the results, SE (diopter) had a positive correlation with the VD (%) values of the SCP outer ring (superior, nasal, and temporal) and SCP inner ring superior. There was also a negative correlation between AL and the FAZ CI and SCP outer ring superior VD (%) values.

## Discussion

Recently, OCTA has been used as a noninvasive technology that allows for the assessment of retinal microvascularity. It was developed by taking sequential structural and cross-sectional B-scan images from a point of the retina in a very short time and converting very small tissue changes, such as erythrocyte flow into decorrelation signals with special software. In this modality, the examination of multimodal images obtained simultaneously has made it possible to achieve a more accurate and detailed diagnosis based on the characteristics of structures and current signal information.^6,7^ Optical coherence tomography angiography is a convenient and an easy method to perform even in children, since it does not involve the use of dye injections, can be performed in a short time, and has almost no side effects.^8^ Optical coherence tomography angiography also provides more detailed information on the vascular structure of the retina compared to OCT. Many studies have been undertaken using OCTA, but to our knowledge, no study has evaluated patients with school-age myopia in comparison with healthy volunteers of similar ages.

We investigated changes in retinal vascular density in patients with simple myopia (˂ 6 diopter) using OCTA and evaluated whether there was any difference compared to age-matched healthy cases. Our results demonstrated reduced values for the VD of SCP and FAZ parameters in the simple myopia group compared to the control group, although the 2 groups had similar ETDRS thicknesses. We also detected a significantly thinner inferior GCC in the simple myopia group compared to the control group. We found that as the AL and SE values increased, the VD of SCP also decreased.

Although the literature contains OCTA findings in healthy children, data on school-age children with simple myopia are limited.^8,9^ Gołębiewska et al^9^ showed that the superficial retinal VD was lower, and the FAZ area was expanded in myopic children compared to emmetropic subjects. Although our study similarly revealed a decrease in the VDs of the SCP outer and inner rings, we detected a smaller FAZ area in the simple myopia group. Moreover, Linderman and Sampson^10,11^ showed that ocular magnification and AL variation could lead to errors in FAZ and VD measurements.

In our study, the VD of SCP had a positive correlation with SE and a negative correlation with AL. Previous studies have shown that decreased superficial and deep VDs in the macular region are associated with longer AL and higher SE values in myopic eyes with different refractive states.^12,13^ Our results were consistent with these studies reporting retinal microvascular network loss in myopia.

Ucak et al^14^ found the GCC (superior and inferior) and ETDRS thickness values to be much lower in young adults with high myopia than in the control group. Although our study showed a lower inferior GCC thickness in the patients with simple myopia compared to the controls, the superior GCC and ETRDS thicknesses were similar in both groups. These discrepancies can be attributed to the differences in the age and myopia degrees of the subjects included in studies.

In the literature, many studies have shown that high myopia causes vascular changes (especially in VD and FAZ parameters) in the retina.^14-16^ In this study, unlike the literature, vascular changes in the macular region of simple myopia were examined and compared with those of the healthy age-matched volunteers. In addition, we investigated whether vascular changes in simple myopia had a relationship with AL and SE.

The most important feature of this study is that it is one of the first in the literature to compare the OCTA findings of simple myopia cases and healthy pediatric controls. On the other hand, our study had certain limitations. First, since the device’s software automatically measured the FAZ and VD values, due to the use of updated device software in the current study, objectivity in comparing results with prior research was reduced. Second, the instrument’s software only allowed for the calculation of VD metrics at the SCP level. Lastly, the sample size was small, and there is a need for a larger number of cases to better evaluate measurements.

In conclusion, this study provided valuable data concerning changes in vessels in the macular region of the retina in school-aged children with simple myopia and confirms the findings of a previous study evaluating a patient population with high myopia. Contrary to the common view, our results showed that there might be changes in the macula in simple myopia. We consider that in the coming years, the frequency of myopia and associated retinal pathologies related to myopia will gradually increase. Therefore, necessary precautions should be taken. Future studies can be carried out with larger sample groups using more advanced devices and software.

## Figures and Tables

**Table 1.** Demographic Features of the Study Participants

	Simple Myopia	Control	*P*
Age (years)	15.36 ± 1.77	15.16 ± 1.87	.55
Male/female	20/14	22/12	.22

**Table 2.** Ocular and OCT Findings of the Study Participants

	Simple Myopia		Control		*P*
	Mean ± SD	Min/Max	Mean ± SD	Min/Max	
SE (diopter)	−2.15 ± 1.40	−0.50/−5.75	0.30 ± 0.29	0.00/1.00	<.001
AL (mm)	24.22 ± 1.14	21.98/26.72	23.33 ± 0.73	22.06/24.98	.001
GCC superior (µm)	109.67 ± 7.82	93.0/127.0	112.83 ± 22.82	93.0/212.0	.928
GCC inferior (µm)	101.61 ± 16.29	37.0/124.0	108.47 ± 9.47	75.0/124.2	.038

AL, axial length; GCC, ganglion cell complex; OCT, optical coherence tomography; SD, standard deviation; SE, spherical equivalent.

**Table 3.** Macular Thickness Measurements of the Study Participants

		Simple Myopia		Control		*P*
		Mean ± SD	Min/Max	Mean ± SD	Min/Max	
ETDRS outer ring thickness (6 mm) (µm)	Superior	328.74 ± 17.02	296.0/379.0	328.22 ± 15.73	298.0/357.0	0.910
	Nasal	337.93 ± 14.22	312.0/382.0	313.72 ± 73.79	0.7/361.0	.281
	Inferior	326.00 ± 15.40	298.0/367.0	328.09 ± 23.02	281.0/359.0	.710
	Temporal	317.40 ± 16.49	291.0/360.0	325.65 ± 23.42	272.0/395.0	.138
ETDRS inner ring thickness (3 mm) (µm)	Superior	345.10 ± 15.99	303.0±385.0	342.74 ± 14.55	314.0/369.0	.582
	Nasal	341.57 ± 17.83	275.0/376.0	339.96 ± 18.61	301.0/389.0	.086
	Inferior	341.40 ± 12.76	319.0/378.0	330.57 ± 23.28	259.0/360.0	.104
	Temporal	326.40 ± 20.78	253.0/361.0	329.78 ± 20.66	301.0/395.0	.872
CMT		268.00 ± 2035	229.0/312.0	266.13 ± 33.68	229.0/373.0	.202

CMT, central macular thickness; ETDRS, Early Treatment Diabetic Retinopathy Study (macular thickness map) score; SD, standard deviation.

**Table 4.** OCTA Findings of the Study Participants

	Simple Myopia		Control		*P*
	Mean ± SD	Min/Max	Mean ± SD	Min/Max	
FAZ area (mm^2^)	0.34 ± 0.61	0.04/0.66	0.44 ± 0.18	0.17/0.74	.038
FAZ peri (mm)	3.11 ± 0.91	1.17/4.63	2.97 ± 0.98	0.99/5.59	.588
FAZ CI	0.43 ± 0.11	0.24/0.65	0.50 ± 0.12	0.29/0.75	.022
SCP outer super VD (%)	17.63 ± 4.72	2.0/23.0	21.00 ± 1.28	19.0/24.0	.004
SCP outer nas VD (%)	17.03 ± 5.14	0.0/22.0	19.96 ± 1.49	18.0/23.0	.037
SCP outer inf VD (%)	17.47 ± 6.42	1.0/23.0	19.35 ± 4.35	1.0/23.0	.495
SCP outer tem VD (%)	16.40 ± 4.90	1.0/21.0	18.74 ± 1.48	15.0/21.0	.112
SCP inner sup VD (%)	11.40 ± 5.73	0.0/21.0	15.00 ± 4.20	6.0/21.0	.014
SCP inner nas VD (%)	10.53 ± 5.35	0.0/18.0	12.91 ± 3.03	4.0/19.0	.046
SCP inner inf VD (%)	11.50 ± 5.45	0.0±20.0	12.22 ± 4.33	1.0/18.0	.607
SCP inner tem VD (%)	10.63 ± 5.48	0.0/20.0	12.22 ± 3.12	4.0/18.0	.191
Central VD (%)	2.00 ± 2.42	0.0/8.0	2.43 ± 3.45	0.0/14.0	.703

CI, circularity index; FAZ, foveal avascular zone; inf, inferior; nas, nasal; peri, perimeter; SCP, superficial capillary plexus; SD, standard deviation; sup, superior; tem, temporal; VD, vessel density.

**Table 5.** Correlations Between SE and AL Values with OCTA Findings

	SE		AL	
	r	*P*	r	*P*
FAZ area (mm^2^)	0.266	.054	−0.227	.102
FAZ peri (mm)	−0.051	.715	−0.189	.176
FAZ CI	0.224	.107	−0.341	.012
SCP outer sup VD (%)	0.477	<.001	−0.302	.028
SCP outer nas VD (%)	0.317	.021	−0.142	.311
SCP outer inf VD (%)	0.144	.305	−0.096	.494
SCP outer tem VD (%)	0.303	.027	−0.255	.065
SCP inner sup VD (%)	0.323	.018	−0.176	.209
SCP inner nas VD (%)	0.258	.062	−0.037	.793
SCP inner inf VD (%)	0.094	.504	0.179	.200
SCP inner tem VD (%)	0.200	.151	−0.101	.472
Central VD(%)	0.089	.527	0.234	.091

AL, axial length; CI, circularity index; FAZ, foveal avascular zone; nas, nasal; inf, inferior; peri, perimeter; SCP; superficial capillary plexus, SE, spherical equivalent; sup, superior; tem, temporal; VD, vessel density.

## References

[1] Wang T Li H Zhang R Yu Y Xiao X Wu C . Evaluation of retinal vascular density and related factors in youth myopia without maculopathy using octa. Sci Rep. 2021;11(1):15361. (10.1038/s41598-021-94909-8); Holden BA Fricke TR Wilson DA et al. Global prevalence of myopia and high myopia and temporal trends from 2000 through 2050. Ophthalmology. 2016;123(5):1036 1042. () PMC8319333 34321564

[2] Morgan IG French AN Ashby RS et al. The epidemics of myopia: aetiology and prevention. Prog Retin Eye Res. 2018;62:134 149. (10.1016/j.preteyeres.2017.09.004) 28951126

[3] De Carlo TE Romano A Waheed NK Duker JS . A review of optical coherence tomography angiography (OCTA). Int J Retina Vitreous. 2015;1:5. (10.1186/s40942-015-0005-8) 27847598 PMC5066513

[4] Campbell JP Zhang M Hwang TS et al. Detailed vascular anatomy of the human retina by projection-resolved optical coherence tomography angiography. Sci Rep. 2017;7:42201. (10.1038/srep42201) PMC5301488 28186181

[5] Early treatment diabetic retinopathy study design and baseline patient characteristics. ETDRS report number 7. Ophthalmology. 1991;98(5)(suppl):741 756. (10.1016/s0161-6420(13)38009-9) 2062510

[6] Li M Yang Y Jiang H et al. Retinal microvascular network and microcirculation assessments in high myopia. Am J Ophthalmol. 2017;174:56 67. (10.1016/j.ajo.2016.10.018) 27818204 PMC5253241

[7] Gao SS Jia Y Zhang M et al. Optical coherence tomography angiography. Invest Ophthalmol Vis Sci. 2016;57(9):OCT27 OCT36. (10.1167/iovs.15-19043) 27409483 PMC4968919

[8] İçel E Yılmaz H Uçak T Taşlı NG Uğurlu A Karakurt Y . Evaluation of the optic disc and macula in healthy children using optical coherence tomography angiography. Turk J Ophthalmol. 2020;50(4):228 233. (10.4274/tjo.galenos.2020.85282) 32854467 PMC7469897

[9] Gołębiewska J Biała-Gosek K Czeszyk A Hautz W . Optical coherence tomography angiography of superficial retinal vessel density and foveal avascular zone in myopic children. PLoS One. 2019 ;14(7):e0219785. (10.1371/journal.pone.0219785) 31318910 PMC6639003

[10] Linderman R Salmon AE Strampe M Russillo M Khan J Carroll J . Assessing the accuracy of foveal avascular zone measurements using optical coherence tomography angiography: segmentation and scaling. Transl Vis Sci Technol. 2017;6(3):16. (10.1167/tvst.6.3.16) PMC5469394 28616362

[11] Sampson DM Gong P An D et al. Axial length variation impacts on superficial retinal vessel density and foveal avascular zone area measurements using optical coherence tomography angiography. Invest Ophthalmol Vis Sci. 2017;58(7):3065 3072. (10.1167/iovs.17-21551) 28622398

[12] Fan H Chen HY Ma HJ et al. Reduced macular vascular density in myopic eyes. Chin Med J (Engl). 2017;130(4):445 451. (10.4103/0366-6999.199844) 28218219 PMC5324382

[13] Liu M Wang P Hu X et al. Myopia-related stepwise and quadrant retinal microvascular alteration and its correlation with axial length. Eye. 2021;35(8):2196 2205. 33087885 10.1038/s41433-020-01225-y PMC8302696

[14] Ucak T Icel E Yilmaz H et al. Alterations in optical coherence tomography angiography findings in patients with high myopia. Eye (Lond). 2020;34(6):1129 1135. (10.1038/s41433-020-0824-1) 32094474 PMC7253420

[15] Jiang Y Lou S Li Y Chen Y Lu TC . High myopia and macular vascular density: an optical coherence tomography angiography study. BMC Ophthalmol. 2021;21(1):407. (10.1186/s12886-021-02156-2) PMC8626942 34836532

[16] Min CH Al-Qattan HM Lee JY Kim JG Yoon YH Kim YJ . Macular microvasculature in high myopia without pathologic changes: an optical coherence tomography angiography study. Korean J Ophthalmol. 2020;34(2):106 112. (10.3341/kjo.2019.0113) 32233143 PMC7105786

